# Acceleration of antibacterial activity of curcumin loaded biopolymers against methicillin‐resistant *Staphylococcus aureus*: Synthesis, optimization, and evaluation

**DOI:** 10.1002/elsc.202100050

**Published:** 2021-12-13

**Authors:** Hamoun Anbari, Amir Maghsoudi, Mohammadreza Hosseinpour, Fatemeh Yazdian

**Affiliations:** ^1^ Department of Food Science and Technology Science and Research Branch, Islamic Azad University Tehran Iran; ^2^ Persisgen Pharmaceutical Accelerator Company Tehran Iran; ^3^ Department of Life Science Engineering Faculty of New Science and Technologies University of Tehran Tehran Iran

**Keywords:** curcumin, methicillin, nanocurcumin, *Staphylococcus aureus*

## Abstract

Curcumin is a polyphenolic molecule with antibacterial, antioxidant, anti‐inflammatory, and antimicrobial properties. This study aimed to prepare nanocurcumin by encapsulating in biopolymers to improve its stability, bioavailability, water‐solubility, antibacterial efficiency against methicillin‐resistant *Staphylococcus aureus*. Three effective variables of curcumin concentration, polymer concentration, and water volume on curcumin‐loaded polymer nanoparticles, were optimized. The average size of polyacrylic acid (PAA), polyvinyl alcohol (PVA), and polyethyleneimine (PEI) nanoparticles were obtained 75.2, 77.1, 86.4 nm, respectively. The nanoparticles had a spherical shape, a smooth and uniform surface morphology. The MIC of PAA, PVA, and PEI nanoparticles was 0.480, 0.390, and 0.340 mg/mL, respectively and the MIC of PAA, PVA, and PEI combined with methicillin was 0.330, 0.260, and 0.200 mg/mL, respectively. According to the results, curcumin‐loaded PEI nanoparticles had the highest inhibitory effect against methicillin‐resistant *S. aureus* among the synthesized nanoparticles. The results showed that solvent volume, polymer concentration and curcumin concentration had a significant effect on particle size. The inhibitory properties of curcumin nanoparticles significantly increased due to the smaller particle size and increased penetration into the bacterium. Curcumin‐loaded nanoparticles can be promising drug carriers for the treatment of infections, cancer, and other diseases.

**Abbreviation**
PAA: polyacrylic acid
PEI: polyethyleneimine
PVA: polyvinyl alcohol
*S. aureus*
: *Staphylococcus aureus*

## 1 INTRODUCTION

Today, the growing prevalence of antibiotic resistance to *Staphylococcus aureus* is one of the problems that physicians are dealing with. *S. aureus* has been known for many years as one of the most important human pathogens. According to the World Health Organization, four of the ten most common causes of death in developing countries are infectious diseases [1]. *S. aureus* is one of the most important and common causes of nosocomial infections worldwide, causing a large number of human infections, including pneumonia, endocarditis, and meningitis. The emergence of antibiotic‐resistant strains is now considered as one of the challenges in treating patients with infections caused by these strains [2]. *S. aureus* is an opportunistic pathogen and often causes asymptomatic disease. Methicillin‐resistant *S. aureus* strains (MRSAs) are dangerous pathogens that are resistant to most of the common antibiotics and can challenge specific treatment of disease [3].

Studies have shown that patients with MRSA infection are hospitalized longer than those infected by methicillin‐sensitive strains or MSSA, and the death rate of patients with MRSA is twice the patients with MSSA [4, 5]. The development of bacterial resistance against existing antibiotics has increased research on the discovery of new plant‐based antibacterial agents. Curcumin (1,7‐bis(4‐hydroxy‐3‐methoxyphenyl)‐1,6‐heptadiene‐3,5‐dione) is a phenolic and non‐polar compound from the root of the *Curcuma longa* plant that has been used for centuries to cure many diseases [6]. Extensive research over the past five decades has shown that curcumin has strong antioxidant, antibacterial, antifungal, antiviral, antimicrobial, anticancer, antidepressant, neuroprotective, tissue protective, metabolic, and immune system regulatory effects [7, 8]. The World Health Organization considers the use of curcumin to be risk‐free [9]. Curcumin has a high ability to inhibit the growth of methicillin‐resistant *S. aureus*. The potential intracellular mechanism and reactive oxygen species (ROS) of the bacterium inhibition of curcumin is mediated by the damage to the bacterial membrane and its permeability, resulting in the death of *S. aureus* [10]. ROS is a phrase used to describe a number of reactive molecules and free radicals derived from molecular oxygen.

Although the widespread availability of curcumin, its safety, low cost, and potential for the prevention or treatment of cancer and other chronic diseases have attracted its development as a drug, but due to its low solubility in water and bioavailability, in clinical trials, good results cannot be obtained.

**PRACTICAL APPLICATION**

- Development of a new method for the production and optimization of curcumin‐loaded nanoparticles.
- Improvement in stability, bioavailability, and aqueous solubilityof curcumin incretion.
- Compared to PVA and PEI nanoparticles containing curcumin, curcumin‐loaded PAA nanoparticle had the smallest size, highest degree of stability in colloidal environments, and the highest level of inhibition against methicillin‐resistant *S. aureus* bacteria.

In recent years, various strategies have been developed to increase the efficacy of curcumin [11]. One of the methods to increase the stability and solubility of curcumin is the use of curcumin nanoformulations [12].

Nanocurcumin is a highly potent antimicrobial agent obtained from turmeric extract and has been known for centuries as a home remedy for many diseases. Drug delivery nanosystems have useful capabilities that did not have that feature before being converted to nanodrugs [13]. PAA, PVA [14], and PEI [15] are environmentally friendly and biodegradable polymers that are suitable carriers for drug delivery to the target cell. The natural compound, curcumin, could be entrapped in these polymers and then be released with a fixed rate through the destruction of the polymer matrix [16, 17, 18]. Nazari‐Vanani et al. used a self‐assembly nanoemulsion drug release system to increase the bioavailability of curcumin. In this study, the maximum concentration of the drug in the blood was increased by 3.95 times and the bioavailability by 1.94 times [19]. In a study by Teow et al., by examining the effect of curcumin on *S. aureus*, they demonstrated its antibacterial effects by disrupting bacterial wall, increasing sensitivity to beta‐lactam antibiotics, inhibiting cytokines, and cell proliferation [20]. In another study by Lian et al., Gold nanoparticles coated with hydroxypropyl beta cyclodextrin were used for curcumin loading. Here, the concentration of nanoparticles was four‐fold compared to the drug [21].

Tyagi et al. studied some gram‐positive bacteria (*S. aureus* and *Enterococcus faecalis*) and gram‐negative bacteria (*Escherichia coli* and *Pseudomonas aeruginosa*). The results showed that the bactericidal effect of curcumin is through damage to the membrane [22]. Aimee et al. investigated the antimicrobial and wound healing effects of the curcumin encapsulated nanoparticles. Based on the results, curcumin‐loaded nanoparticles were able to inhibit the growth of methicillin‐resistant *S. aureus* (MRSA) and *P. aeruginosa* in dose‐dependent laboratory conditions [23]. Negi et al. investigated the inhibitory effect of efflux pumps (PABN) and curcumin on drug‐resistant *P. aeruginosa* isolates. The results show that curcumin is able to have a similar effect to this drug. Therefore, it was stated that curcumin might act as an inhibitor of efflux pumps in *P. aeruginosa* [24].

The aim of the present study was to synthesize and optimize PAA, PVA, and PEI nanoparticles containing curcumin and methicillin antibiotics and then evaluate their inhibitory effects against methicillin‐resistant *S. aureus*.

These days, numerous novel medicines are suggested as options to conventional methods. Between them, controlled drug delivery systems with lowest side effects are viewed as predominant. These systems are qualified for drug release in particular places of the body. Three parts of the controlled delivery systems are therapeutic agent, targeting section, and carrier matrix. The therapeutic agents may be categorized as biologicals, like curcumin, and non‐biologicals [25, 26, 27].

## 2 MATERIALS AND METHODS

In this study, in order to evaluate the efficiency of curcumin nanoparticles, *S. aureus* PTCC 1764 purchased from the Scientific and Industrial Research Organization of Iran was used. The bacterium was cultured in Luria‐Bertani (LB) culture medium (Merck, Germany) containing 1% peptone, 0.5% yeast extract, 1% sodium chloride with 1% glucose for 18 h at 37°C. Curcumin, PAA, PVA, and PEI were purchased from Sigma Aldrich. LB medium, glycerol and glucose were obtained from Merck, Germany.

### 2.1 Statistical optimization of drug loading in polymer nanoparticles

In this study, the concentrations of three polymers of PAA, PVA, and PEI (A), the concentration of curcumin (B) and the volume of solvent (C) as the three variables affecting the loading efficiency were selected to be investigated. In order to optimize these three factors, the response level method was used. In order to screen and evaluate important indicators at maximum load efficiency, the Design Expert software was used. The variables in this statistical optimization method were studied at five levels. Various levels for optimizing curcumin loading and particle size are listed in Table 1.

**TABLE 1:** Variable levels in statistical optimization of curcumin loading in polymer nanosystems

Sign	Variable	Low surface (Code ‐1)	Central surface (Code 0)	High surface (Code +1)	Actual value of code –1.68	Actual value of code +1.68
**A**	Curcumin concentration (mg/mL)	0.49	1.05	1.61	0.1	2
**B**	Polymer concentration (mg/mL)	0.49	1.05	1.61	0.1	2
**C**	Water volume (mL)	0.28	0.55	0.82	0.1	1

### 2.2 Synthesis of curcumin‐loaded polymer nanoparticles

In order to determine the loading efficiency of the drug in the polymer nanoparticles synthesized by nanoprecipitation method (PAA, PVA, and PEI), a standard calibration curve for curcumin was prepared. The drug loading and its concentration in nanoparticles was determined by measuring the optical density using a spectrophotometer at the wavelength of 422 nm [28].

For the synthesis of nanoparticles, first different concentrations of three polymer (0.2–1 μg/mL), PAA, PVA, and PEI as well as different concentrations of curcumin (0.2–1 μg/mL) and volumes of solvent (0.1–1 ml) were considered as variables and were optimized by performing multiple experiments. To synthesize PAA nanoparticles, 0.49 μg of PAA was dissolved in 0.28 ml water using a magnetic stirrer at 500 rpm, then 1.61 μg dissolved curcumin in acetone was added dropwise to the aqueous solution of PAA and stirred for 1 h. After a few minutes, the sedimentation of the nanoparticles was observed at the bottom of the glass container, and in order to evaporate the acetone, the nanoparticle solution was stirred on a magnetic stirrer for 12 additional hours. At the end, to separate the nanoparticle sediment, an ultrasonic bath (340 W) was used for 10 min at room temperature. In the next step, the content of the glass container along with the PAA nanoparticles was filtered using a suitable syringe filter and it was transferred to Falcon and kept in the refrigerator. For the synthesis of PVA and PEI nanoparticles, it was determined that 1.61 μg of PVA and 1.61 μg of PEI dissolved in 0.28 ml of water were the optimum values to obtain the highest curcumin loading. The rest of the procedure was similar to the synthesis of PAA nanoparticles.

### 2.3 Characterization of nanoparticles

Surface morphology of nanoparticles (roughness, shape, smoothness, and aggregation) was observed using SEM (Scanning electron microscope) device. In order to investigate the surface charge and stability of the nanoparticles containing curcumin, the zeta potential was measured using Brookhaven Instruments Corp.’s Zeta Sizer device. Dynamic Light Scattering (DLS) measurement of nanoparticles was performed at the angle of 90°, the wavelength of 657 nm and 25°C. Each sample was diluted to 0.1 mg/mL and measured immediately after preparation [29].

### 2.4 Determination of MIC of curcumin‐loaded polymer nanoparticles on *S. aureus*

Micro dilution method was used to determine the minimum inhibitory concentration (MIC) of curcumin‐loaded polymer nanoparticles on methicillin‐resistant *S. aureus*. MIC is defined as the lowest concentration of an antimicrobial that will inhibit the visible growth of a microorganism after overnight incubation. 18‐h bacterial culture was performed at the Luria‐Bertani culture medium at 150 rpm and 37°C. After this time, the OD_600_ of the inoculated material was set to 0.2 by fresh Luria‐Bertani. To perform the assay, a 96‐well plate was used. First, 1 ml of inoculation material was added to each well and volumes of 128, 64, 32, 16, 8, 4, 2, 0 μL of the nanoparticle solution (1 mg/mL) were added. The 96‐well plate was incubated for 24 h at 37°C, and the optical absorption was recorded using an ELISA device at 600 nm. MIC of nanoparticles is equal to the lowest concentration of nanoparticles that results in OD_600_ less or equal to 0.05 [30, 31].

## 3 RESULTS

### 3.1 Characteristics of nanoparticles

**FIGURE 1:**
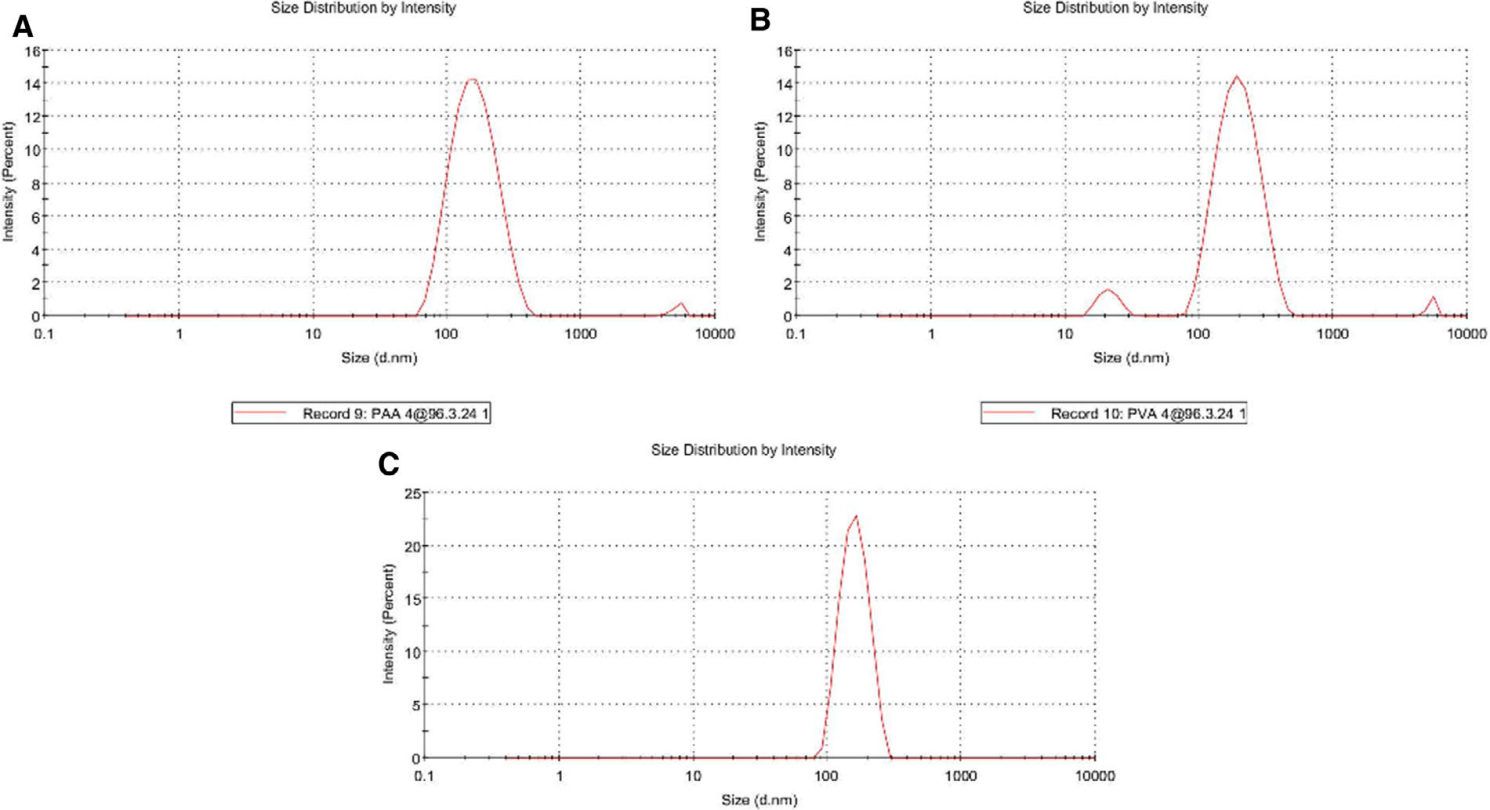
The average size of polymer nanoparticles; (A) curcumin‐loaded PAA nanoparticles, (B) curcumin‐loaded PEI nanoparticles; (C) curcumin‐loaded PVA nanoparticles

The size of polymer nanoparticles was determined using DLS. DLS is a physical method used to determine the distribution of particles in solutions and suspensions. As shown in Figure 1, the average size of curcumin‐loaded PAA, PVA, and PEI nanoparticles was 149.3, 175.8, and 184.5 nm, respectively.

**FIGURE 2:**
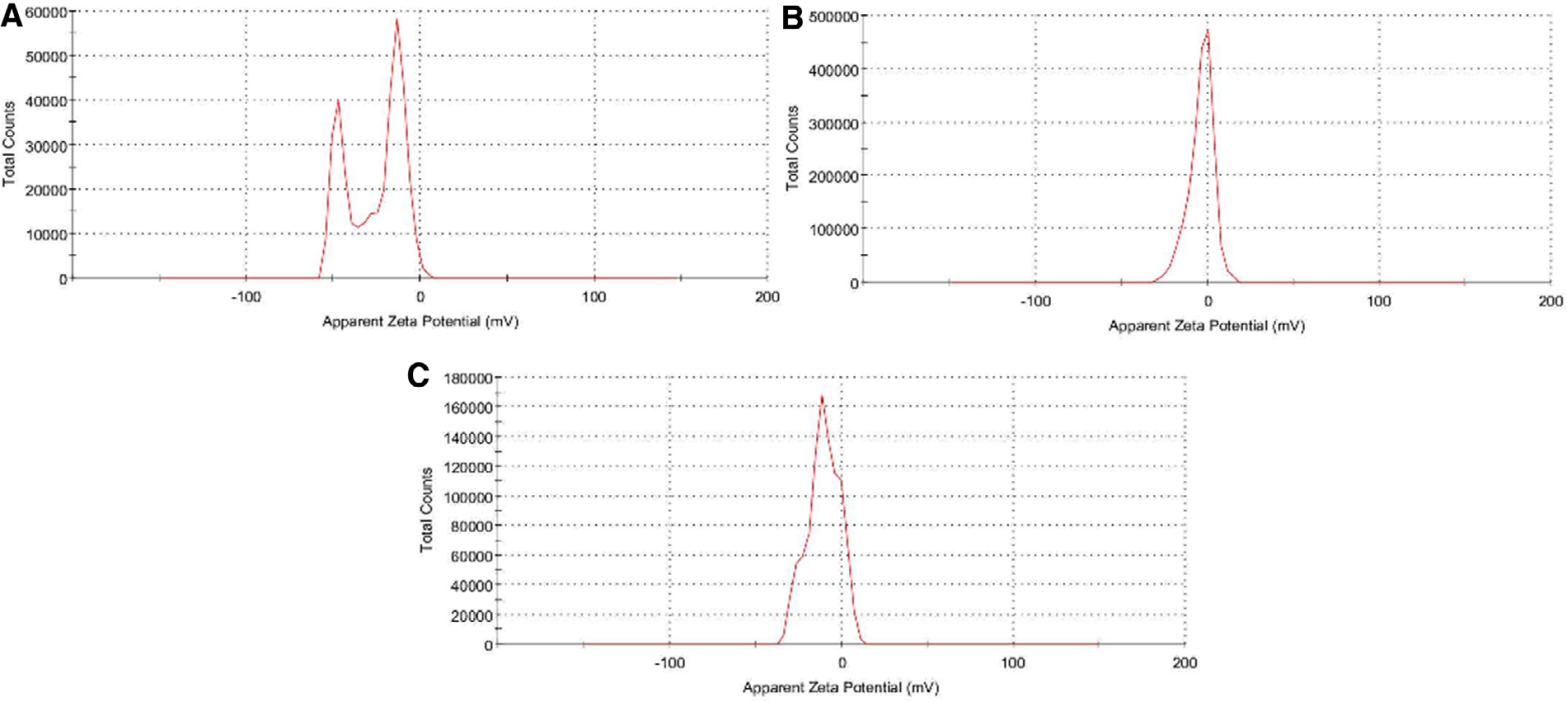
Zeta potential of nanoparticles containing curcumin; (A) PAA containing curcumin, (B) PEI nanoparticles containing curcumin, (C) PVA nanoparticles containing curcumin

According to the results, the aqueous solubility of the nanoparticles containing curcumin significantly increases with decreasing particle size to the nano‐range. The stability of nanoparticles can be confirmed by their potential zeta value. In fact, the zeta potential is an indicator of the potential stability of the colloidal system. In this study, to estimate the surface charge and stability of the nanoparticles containing curcumin, their zeta potential was measured. The results show that the zeta potential of PAA, PVA, and PEI nanoparticles containing curcumin was –26.0, 3.83, and 10.7 mV, respectively (Figure 2). Thus, PAA nanoparticles had the highest stability while PVA nanoparticles had the lowest stability.

Negatively charged nanoparticles possess less cytotoxicity than positively charged nanoparticles and are FDA approved. Of course, both positively and negatively charged nanoparticles have their own advantages, and the surface charge density of nanoparticles should be optimized to have the lowest toxicity and most effective intracellular conductivity [32].

**FIGURE 3:**
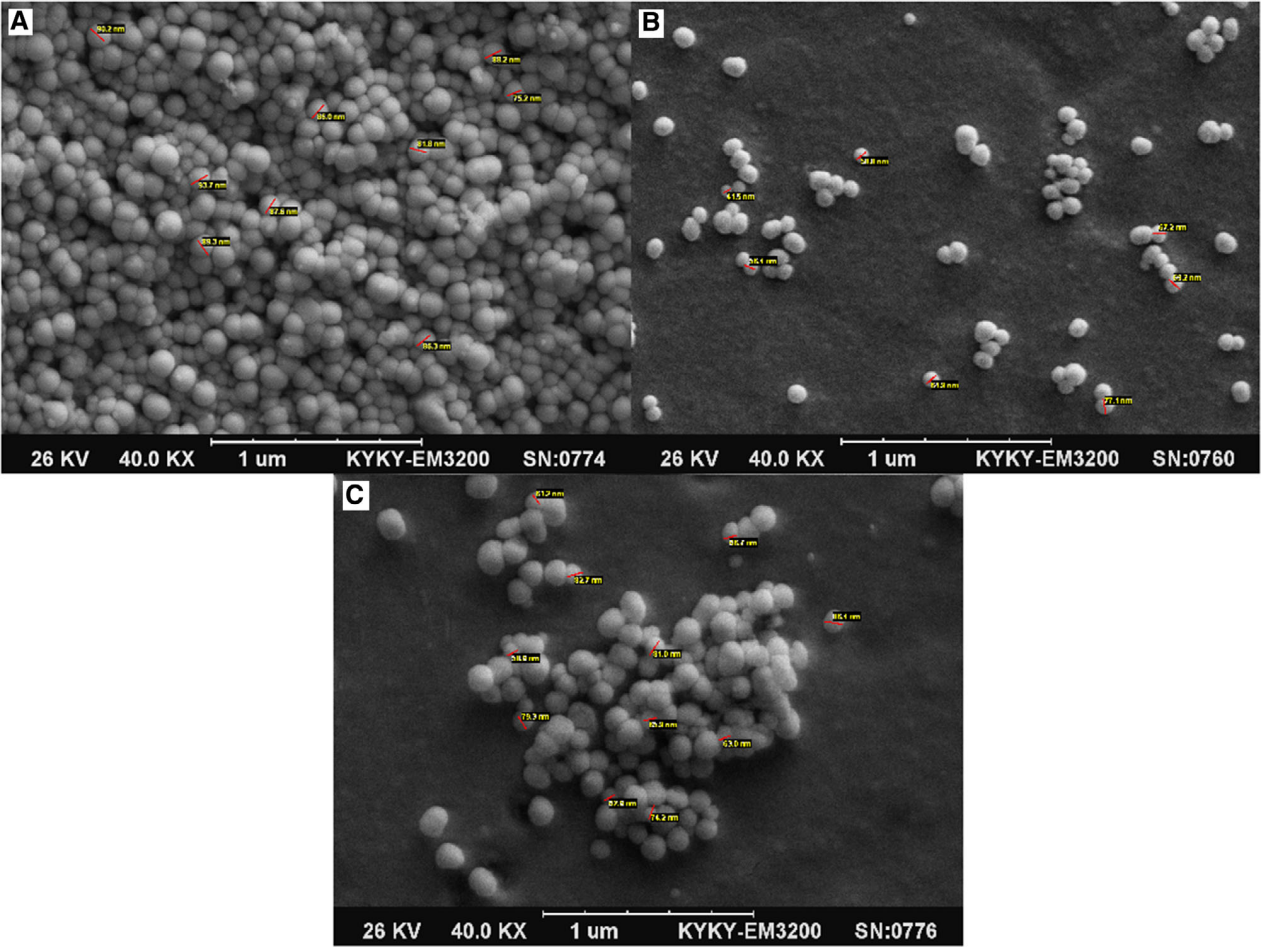
SEM image of nanoparticles containing curcumin: (A) PAA nanoparticles, (B) PVA nanoparticles, (C) PEI nanoparticles

Figure 3 shows SEM images of PAA nanoparticles containing curcumin (A), PVA nanoparticles containing curcumin (B), and PEI nanoparticles containing curcumin (C). SEM images showed that all curcumin‐loaded nanoparticles had spherical shape and dense structure with a smooth and uniform surface. The size of PAA, PVA, and PEI nanoparticles containing curcumin was observed to be 75.2, 77.1, 86.4 nm, respectively. In fact, DLS measures the hydrodynamic radius in the suspension solution and is larger, but SEM shows the particle size in the dry state, which is smaller than the nanoparticle size measured by DLS [33].

### 3.2 Optimization of effective factor in the procedure of curcumin‐loaded polymer nanoparticles synthesis in order to increase the loading efficiency using RSM method

Curcumin concentration (variable A), polymer concentration (variable B), and volume of solvent (variable C) are among the formulation variables influencing the characteristics of curcumin‐loaded polymer nanoparticles specially loading efficiency [34]. The variables were examined for all three nano systems and the responses were evaluated separately. In order to optimize these three factors, the response surface method was used and a response was determined for each experiment. The R_1_ response is related to the concentration of loaded curcumin (μg/mL) determined by optical absorption at 422 nm. According to the design of the experiment for three variables, 17 experiments (14 experiments and three repetitions) were determined. Five levels were considered for the range of levels of variables, including the lower level (code 1‐), central level (zero code), high level (code 1 + 1), code ‐1.68 and code +1.68 (Table 2).

**TABLE 2:** Design table of test levels of response and obtained responses for the curcumin‐loaded nanoparticles

Experiment	Curcumin concentration (Variable A)	Polymer concentration (Variable B)	Water volume (Variable C)	Concentration of loaded Curcumin in PAA nanoparticles (mg/mL)	Concentration of loaded Curcumin in PVA nanoparticles (mg/mL)	Concentration of loaded Curcumin in PEI nanoparticles (mg/mL)
1	1	1	1	270	234	236
2	1	1	1	300	273	241
3	1	1	1	262	265	240
4	1	1	1	261	279	307
5	1	1	1	273	115	141
6	1	1	1	180	165	225
7	1	1	1	156	113	176
8	1	1	1	143	196	217
9	1.68	0	0	150	226	190
10	1.68	0	0	214	276	282
11	0	1.68	0	240	262	156
12	0	1.68	0	176	198	280
13	0	0	1.68	256	266	293
14	0	0	1.68	134	170	185
15	0	0	0	257	178	267
16	0	0	0	289	185	278
17	0	0	0	254	232	279

### 3.3 Data analysis of PAA nanoparticles by response surface method

According to the responses obtained from the Design Expert software, variable A is the concentration of curcumin, variable B is the concentration of PAA polymer and variable C is the volume of water. Also, the R1 response is related to the concentration of loaded curcumin in PAA nanoparticles (μg/mL).

### 3.4 Variance decomposition of PAA nanoparticle

Tables 3 and 4 show the results obtained from the analysis of variance of effects of three variables A, B, and C on the loading of curcumin in PAA nanoparticles.

**TABLE 3:** Software indicators of optimization of curcumin loading efficiency in PAA Nanoparticles

Phrase	Sum of squares	Mean square	F value	*p‐*value Prob > F
A	68.72	68.72	0.41	0.8438
B	6974.92	6974.92	4.15	0.0689
C	21843.333	21843.333	13.00	0.0048
AB	300.12	300.12	0.18	0.6815
AC	2278.13	2278.13	1.36	0.2713
BC	1431.13	1431.13	0.85	0.3778

R‐Squared = 0.6619.

**TABLE 4:** Optimal conditions of maximum loading of curcumin in PAA nanoparticles

No.	Curcumin	PAA	Water	Loading amount	Satisfaction
1	1.61	0.49	0.28	286.622	0.919
2	1.61	0.49	0.28	286.513	0.919
3	1.61	0.51	0.28	286.459	0.918

The volume of water (ml). Satisfaction amount of satisfaction.

**FIGURE 4:**
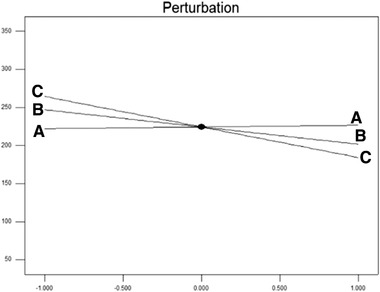
The trend of the effect of independent variables on the dependent variable (loading efficiency) (horizontal axis indicates a deviation from the reference point (coded points))

The results of these tables show that only the main effect of water volume variable (variable C) is significant at the level of 1%, and none of the other main effects along with the interaction effects are significant. Also, in this software, *p* < 0.05 is considered as the level of assurance of the effectiveness of the coefficient and the *p*‐value for the water volume variable is less than 0.05, so this term is valuable in the second degree model. Therefore, the concentration of curcumin loaded in the nanoparticles (R1 response) is a function of this term. Figure 4 shows the trend of the effect of independent variables on the dependent variable (loading efficiency) in perturbation diagram.

**FIGURE 5:**
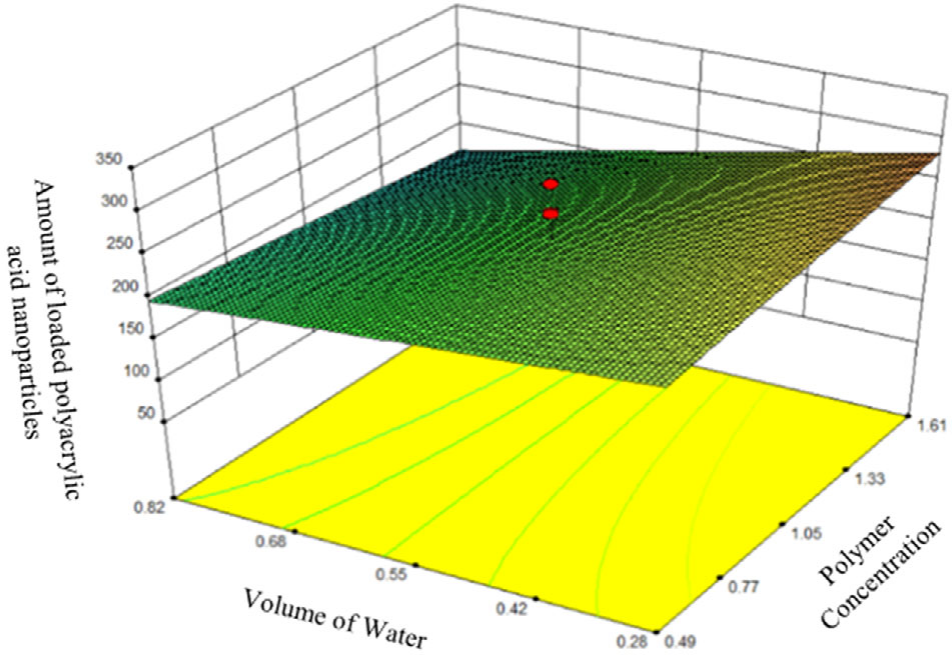
3D image of the interaction of polymer concentration (B) and water volume (C) on the loading efficiency of PAA nanoparticles

According to Figure 4 and the ANOVA table, the highest effect is related to factor C (water volume). The results showed that with increasing water volume, the loading of curcumin in PAA nanoparticles decreased. Increasing the polymer concentration reduced the loaded efficiency as well, but increasing the curcumin concentration slightly increased in the loading efficiency. It should be noted that the effect of the two variables of polymer and curcumin concentration was not statistically significant at the level of 5%. Figure 5 shows a three‐dimensional image of the interaction of polymer concentration (B) and water volume (C) on the loading efficiency of PAA nanoparticles.

Figure 5 shows that at high levels of water volume (0.82), increasing the polymer concentration leads to a decrease in the loading of curcumin but at low levels of water volume (0.28), increasing the polymer concentration enhances the loading efficiency. The response level method presented the following model as the proposed model with a coefficient of determination of 0.66 according to the actual values of the independent variables.

The loading amount of curcumin in PAA nanoparticles = 250.03905 + 45.22227 * Cur‐11.49268 * Poly + 60.68060 * A/W + 19.19570 * Cur * Poly ‐ 111.64844 * Cur * A/W‐88.49173 * Poly * A/W

Due to the selection of the maximum value for the dependent variable (the loading amount of curcumin in PAA nanoparticles) and the placement of independent factors in the test domain, the formulations shown in the table were introduced as the optimal formulations.

### 3.5 Data analysis of PVA nanoparticles by response surface method

Data analysis of PVA nanoparticles was performed according to the responses obtained from the Design Expert software using the response, variable A is the concentration of curcumin, variable B is the concentration of PVA polymer and variable C is the volume of water. The R1 response is related to the concentration of loaded curcumin (μg/mL) in PVA nanoparticles.

### 3.6 Variance decomposition of PVA nanoparticles

Table 5 shows the results of variance decomposition of the effect of three variables A, B, and C on the loading of curcumin in PVA nanoparticles.

**TABLE 5:** Software indicators of optimization of loading efficiency in PVA nanoparticles

Term	Sum of squares	Mean square	F value	*p*‐value Prob > F
A	6077.22	6077.22	4.73	0.0547
B	40.90	40.90	0.032	0.8619
C	30128.52	30128.52	23.46	0.0007
AB	84.50	84.50	0.066	0.8027
AC	480.50	480.50	0.37	0.5544
BC	84.50	84.50	0.066	0.8027

R‐Squared = 0.7418.

The results of Table 5 show that only the main effect of the water volume variable (variable C) was significant at the level of 1%, and none of the other main effects along with the interaction effects was significant. Of course, the effect of curcumin was very significant, although at the level of 5%, it was not significant. Also, in this software, *p* < 0.05 is considered as the level of assurance of the effectiveness of the coefficient and the *p*‐value for the water volume variable is less than 0.05, so this term is important in the presented second degree model. Therefore, the concentration of curcumin loaded in the nanoparticles (R1 response) is a function of this term.

**FIGURE 6:**
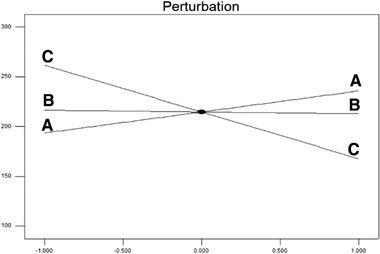
The trend of the effect of independent variables on the loading efficiency of curcumin in PVA nanoparticles (horizontal axis indicating deviation from the reference point (coded points)

Figure 6 shows the trend of the effect of independent variables on the dependent variable (the loading efficiency of curcumin in PVA nanoparticles) in the perturbation diagram. The value of the coefficient of determination in these experiments was 0.74. Typically, R2 above 0.7 indicates a relatively good coefficient of determination, and the closer this value is to one, the greater the compatibility of the experimental data with the regression model, and the higher the accuracy of the model.

According to Figure 6 and the ANOVA table, the maximum effect is related to factor C (water volume). In addition, according to the figure, with increasing water volume, the loading of curcumin in PVA nanoparticles decreased. Furthermore, increasing the curcumin concentration led to an increase in the loading efficiency, but increasing or decreasing the polymer concentration had no effect on curcumin loading in PVA nanoparticles. The response surface method presented the following model as the proposed model with a coefficient of determination of 0.74 according to the actual values of the independent variables.

The loading amount of curcumin in PVA nanoparticles = 303.73973‐1.55188 * Cur‐1.93200 * Poly‐ 206.80053 * A/W + 10.18547 * Cur * Poly + 51.27558 * Cur * A/W ‐ 21.50266 * Poly * A/W

According to the selection of the maximum value for the dependent variable (the loading efficiency of curcumin in PVA nanoparticles) and the placement of independent factors in the test domain, the formulations shown in Table 6 were introduced as the optimal formulations.

**TABLE 6:** Optimal conditions for obtaining the maximum curcumin loading in PVA nanoparticles

No.	Curcumin	PVA	Water	Loading amount	Satisfaction
1	1.61	1.61	0.28	279.848	0.907
2	1.61	1.61	0.28	279.719	0.906
3	1.61	1.60	0.28	279.713	0.906

### 3.7 Data analysis of PEI nanoparticles with response surface method

Data analysis was performed according to the responses obtained from the Design Expert software using the response surface method. It is worth noting that the variable A represents the concentration of curcumin, variable B is the concentration of PEI and variable C is the volume of water. Also, R1 response is related to the concentration of loaded curcumin in PEI nanoparticles (μg/mL).

### 3.8 Variation decomposition of PEI nanoparticles

Table 7 shows the results of variance decomposition of the effects of factors A, B, and C on the loading of curcumin in PEI nanoparticles.

**TABLE 7:** Software indicators of optimization of loading efficiency of curcumin in PEI nanoparticles

Term	Sum of squares	Mean square	F value	*p*‐value Prob > F
A	9058.49	9058.49	17.52	0.0041
B	6835.84	6835.84	13.22	0.0083
C	14606.70	14606.70	28.25	0.0011
AB	45.13	45.13	0.087	0.7762
AC	351.13	351.13	0.68	0.4370
BC	231.13	231.13	0.45	0.5252
A2	2498.27	2498.27	4.83	0.0639
B2	5090.86	5090.86	9.85	0.0164
C2	2154.95	2154.95	4.17	0.0825

**FIGURE 7:**
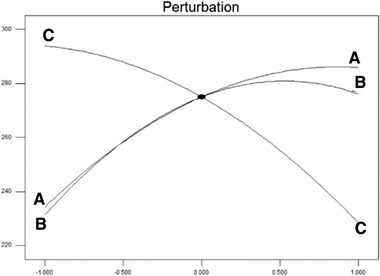
The trend of the effect of independent variables on the loading efficiency of PEI nanoparticles (horizontal axis indicates deviation from the reference point (coded points)

The results of Table 7 show that all the main effects of independent variables on the loading efficiency of PEI nanoparticles at the level of 1% were significant. *p* < 0.05 was considered as the level of assurance of the effectiveness of the coefficient and the *p*‐value for variables A (curcumin concentration), B (polymer concentration), and C (water volume) was less than 0.05. Therefore, these variables are important in the proposed secondary model, so the concentration of curcumin loaded in nanoparticles (R1 response) was a function of this term. The value of the coefficient of determination in these experiments was 0.91. Typically, the high R2 indicates a relatively good coefficient of determination and indicates a good adaptation of the experimental data with the regression model and the high accuracy of the model. Figure 7 shows the trend of the effect of independent variables on the dependent variable (loading efficiency of PEI nanoparticles) in the perturbation diagram.

According to Figure 7 and the ANOVA table, all three factors A, B, and C had a significant effect on the loading efficiency of PEI nanoparticles. As seen in the perturbation diagram, the effect of water volume (variable C) on the loading of curcumin in PEI nanoparticles was reversed, but with increasing curcumin concentration (variable A) and PEI concentration (variable B), the loading efficiency increased. In addition, at lower levels than the intermediate level, an increase in each variable leads to an increase in drug loading, while higher levels of the polymer concentration lead to a gradual decrease in the loading efficiency, while higher concentrations of curcumin, gradually increased the loading efficiency.

**FIGURE 8:**
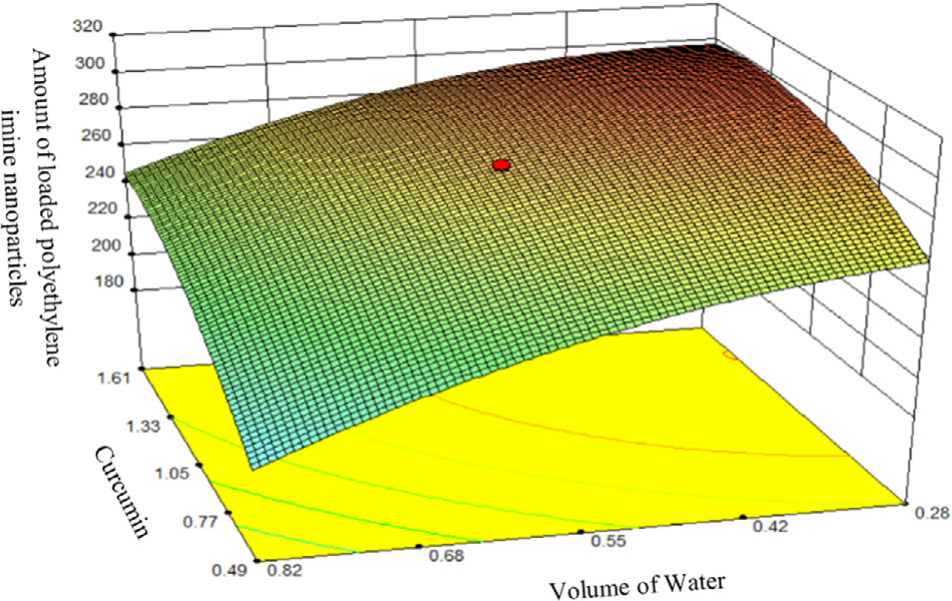
3D image of the interaction effects of curcumin concentration (variable A), polymer concentration (variable B) and water volume (variable C) on the loading efficiency of PEI nanoparticles

Figure 8 shows a three‐dimensional image of the interaction effects of curcumin concentration (variable A), polymer concentration (variable B) and water volume (variable C) on the loading efficiency of PEI nanoparticles.

Based on the results of Figure 8, at low water volume levels (0.28), increasing the curcumin concentration resulted in a small increase in loading efficiency, but with increasing curcumin levels at high water volume levels (0.82), the loading efficiency increased sharply. The response surface method presented the following model as the proposed model with a coefficient of determination of 0.91 according to the actual values of the independent variables.

The loading amount of curcumin in PEI nanoparticles = 82.48790 + 111.64369 * Cur + 191.20774 * Poly + 81.51485 * A/W + 7.44323 * Cur * Poly + 43.83235 * Cur * A/‐ 35.56210 * Poly * A/W ‐ 46.65406 * Cur^2^ ‐ 66.59866 * Poly^2^ ‐ 193.11256 * A/W^2^

Due to the selection of the maximum value for the dependent variable (the loading amount of curcumin in PEI nanoparticles) and the placement of independent variables in the test domain, the formulations shown in the Table 8 were introduced as the optimal formulations.

**TABLE 8:** Optimal conditions for maximum loading of curcumin in PEI nanoparticles

No.	Curcumin	PEI	Water	Loading amount	Satisfaction
1	1.34	1.37	0.30	309.36	1
2	1.27	1.55	0.30	307.295	1
3	1.52	1.28	0.33	307.283	1

### 3.9 Antimicrobial properties of curcumin‐loaded polymer nanoparticles

Table 9 shows the results of MIC test of curcumin‐loaded polymer and pure curcumin on methicillin‐resistant *S. aureus*.

**TABLE 9:** Antimicrobial activity of curcumin‐loaded polymer nanoparticles, pure curcumin, and pure methicillin on methicillin‐resistant *Staphylococcus aureus*

Sample	MIC against Methicillin‐resistant *Staphylococcus aureus* (mg/mL)
PEI	0.340
PVA	0.390
PAA	0.480
Curcumin	0.560
PEI with Methicillin	0.200
PVA with Methicillin	0.260
PAA with Methicillin	0.330
Methicillin	0.300

Nanoparticle antimicrobial properties were tested in three replications. MIC was reported as the lowest concentration at which curcumin‐loaded nanoparticles were able to fully inhibit the growth of the tested bacterium (OD_600_ less than or equal to 0.05) [35].

As shown in Table 9, the minimum MIC of polymer nanoparticles for *S. aureus* without the presence of the methicillin belonged to PEI nanoparticles which was equal to 0.340 mg/mL. The MIC of PVA nanoparticles was 0.390 mg/mL and the MIC of PAA nanoparticles was 0.480 mg/mL. MIC of pure curcumin was 0.560 mg/mL, which was higher than MIC of curcumin‐loaded polymer nanoparticles. But the second experiment was to study the antimicrobial properties of polymer nanoparticles with the methicillin. The MIC of methicillin alone was 0.3 mg/mL. Also, MIC for PEI, PVA and PAA nanoparticles with methicillin was equal to 0.20, 0.26, and 0.330 mg/mL, respectively. According to the results, curcumin‐loaded PEI nanoparticles had the highest inhibition against methicillin‐resistant *S. aureus* among the synthesized nanoparticles.

## 4 DISCUSSION

This study aimed to synthesize, optimize and evaluate the antibacterial effect of curcumin‐loaded polymer nanoparticles against methicillin‐resistant *S. aureus*. Curcumin has anti‐inflammatory, antibacterial, antifungal, antiviral, antimicrobial, wound healing, and antioxidant properties which has the ability to induce apoptosis in many cellular systems [36, 37, 38]. However, due to its low bioavailability and high degradation rate in biological systems, it has no clinical effects [39]. Research on nanomaterials suggests that nanocurcumin has high bioavailability. Particle size has been shown to be an important variable because it can directly affect physical stability, cellular uptake, and drug release from nanoparticles [40]. Nanocurcumin due to its significantly reduced size compared to the size of curcumin has a better penetration and higher uptake by cell or bacteria. Laboratory biometric measurements have also clearly shown that converting to nanomaterial form greatly increases water solubility and antibacterial activity of curcumin [41]. According to the SEM results, the size of curcumin‐loaded PAA, PVA, and PEI nanoparticles was 149.3, 175.8, and 184.5 nm, respectively. Measuring the Zeta potential showed the high stability of synthesized nanoparticles in colloidal environments. The results of the present study showed that polymer nanoparticles containing curcumin could have antibacterial properties against methicillin‐resistant *S. aureus*. It was also found that due to the reduction in the size of curcumin nanoparticles compared to pure curcumin, the MIC of curcumin nanoparticles was lower than that of pure curcumin, as the MIC of PAA, PVA, and PEI nanoparticles was 0.480, 0.39, and 0.34 mg/mL, respectively, while for pure curcumin, it was 0.560 mg/mL.

Studies have shown that nanomaterials linked to antibiotics increase the concentration of antibiotics at the site of infection and facilitate their binding to bacteria. Therefore, the combination of nanomaterials with antimicrobial peptides and essential oils creates a real synergy against bacterial resistance [42]. It has also been shown that curcumin not only has antibacterial properties but also has a synergistic effect with antibiotics. Examination of the synergistic effect of curcumin‐loaded nanoparticles with methicillin in this study showed that the MIC of methicillin alone was 0.3 mg/mL and MIC of curcumin‐loaded PAA, PVA, and PEI nanoparticles combined with the methicillin was 0.33, 0.26, and 0.2 mg/mL, indicating that the inhibitory effect of curcumin‐loaded nanoparticles and the methicillin together is greater than the inhibitory effect of each alone. Mun et al. investigated the synergistic effect of curcumin and the ciprofloxacin, norfloxacin, oxacycline, and ampicillin on methicillin‐resistant *S. aureus*, and stated that the use of curcumin with each of these antibiotics considerably increased the inhibition of bacterial growth [2]. The results of research by Adahoun et al. showed that changing the size of curcumin nanoparticles significantly increased the antibacterial activity of this compound. The MIC of nanocurcumin was much lower than that of pure curcumin for all strains used in the study. Also, due to the increase in the solubility of nanocurcumin compared to pure curcumin, its MIC against bacteria increases [41]. Basniwal et al. also stated that gram‐positive bacteria are more sensitive than gram‐negative bacteria to curcumin‐loaded nanoparticles, which is due to the differences in the cell wall structure of the two groups [7]. Research by Adahoun et al. on strains of gram‐negative *E. coli* and gram‐positive *S. aureus* showed that curcumin‐loaded nanoparticles had the stronger effect on *S. aureus* [41]. A research by Jin et al. also confirmed previous findings that reducing the size of curcumin‐loaded nanoparticles increases its permeability into bacterial wall and its uptake by cells.[42]

## 5 CONCLUDING REMARKS

In the present study, a new method for the production and optimization of curcumin‐loaded nanoparticles was developed to improve stability, increase bioavailability and aqueous solubility of curcumin.

Examination of the antimicrobial effect of PAA, PVA, and PEI nanoparticles containing curcumin showed that due to the reduction in the size of curcumin particles, increased water solubility and increased bacterial uptake, the ability of this substance to inhibit the growth of methicillin‐resistant *S. aureus* increased and these nanoparticles can be safely used to treat infections caused by this bacterium.

Also among the nanoparticles synthesized in this study, curcumin‐loaded PEI nanoparticle had the highest degree of stability in colloidal environments and highest level of inhibition against methicillin‐resistant *S. aureus*.

The results of the study showed that curcumin‐loaded PAA, PVA, and PEI nanoparticles, in addition to increasing the solubility of curcumin, can be considered as promising carriers for drugs that have low water solubility, and with the suitable release of drug molecules into the bloodstream, they can reach the target site and have therapeutically effect. These nanoparticles can also effectively inhibit the growth of antibiotic‐resistant *S. aureus*. Therefore, they can be used as a new strategy to inhibit the growth of bacteria such as *S. aureus*.

## CONFLICT OF INTEREST

The authors have declared no conflict of interest.

## Data Availability

The data that support the findings of this study are available from the corresponding author upon reasonable request.
